# Dual-resonance enhanced quantum light-matter interactions in deterministically coupled quantum-dot-micropillars

**DOI:** 10.1038/s41377-021-00604-8

**Published:** 2021-07-29

**Authors:** Shunfa Liu, Yuming Wei, Xueshi Li, Ying Yu, Jin Liu, Siyuan Yu, Xuehua Wang

**Affiliations:** 1 State Key Laboratory of Optoelectronic Materials and Technologies, School of Physics, School of Electronics and Information Technology, Sun Yat-sen University, Guangzhou, 510275 China; 2 Photonics Group, Merchant Venturers School of Engineering, University of Bristol, Bristol, BS8 1UB UK

**Keywords:** Single photons and quantum effects, Nanocavities

## Abstract

Optical microcavities have widely been employed to enhance either the optical excitation or the photon emission processes for boosting light-matter interactions at the nanoscale. When both the excitation and emission processes are simultaneously facilitated by the optical resonances provided by the microcavities, as referred to the dual-resonance condition in this article, the performances of many nanophotonic devices approach to the optima. In this work, we present versatile accessing of dual-resonance conditions in deterministically coupled quantum-dot (QD)-micropillars, which enables emission from neutral exciton (X)—charged exciton (CX) transition with improved single-photon purity. In addition, the rarely observed up-converted single-photon emission process is achieved under dual-resonance conditions. We further exploit the vectorial nature of the high-order cavity modes to significantly improve the excitation efficiency under the dual-resonance condition. The dual-resonance enhanced light-matter interactions in the quantum regime provide a viable path for developing integrated quantum photonic devices based on cavity quantum electrodynamics (QED) effect, e.g., highly efficient quantum light sources and quantum logical gates.

## Introduction

The last decade has witnessed significant advances in nanophotonics by harnessing the enhanced light-matter interaction in optical microcavities^1^. E.g., cavity-enhanced scattering and excitation enable the realization of biosensing with sensitivity down to the single-molecule level^2–4^ and highly efficient optical harmonic generations^5,6^. On the emission part, microcavities can modify the photonic environments of the nanoscale quantum emitters, resulting in faster radiative emission rate and better far-field radiation directionality^7–10^. However, most of the nanophotonic devices based on high quality (Q) dielectric microcavities, to date, only involves the single resonance condition either for boosting the excitations or improving the photon emissions. Ideally, it is possible and highly desirable to simultaneously enhance both the excitation and the emission processes under the multiple-resonance condition, which is however technologically challenging especially for the dielectric microcavities with high-Q factors. Only until very recently, dual and even triply resonances conditions have been achieved in photonic crystal cavities, micro-rings, and microspheres, which leads to unprecedented device performances including Raman laser^11,12^, frequency conversion^13,14^, surface nonlinear optics^15^ and on-chip optical parametric oscillation with a record low threshold^16^. For single semiconductor QDs, most studies are focused on the enhancements of the emission process to pursue optimal single-photon sources^17–19^. While the cavity-enhanced P-shell excitation^20^, wetting layer excitation^21^, and phonon-assisted excitation^22^ have been observed by utilizing the high-order cavity modes of photonic nanocavities, the dual-resonance enhanced excitation-emission process has not been reported yet. In this work, we present versatile accessing of the dual-resonance conditions in deterministically coupled QD-micropillars operating in the cavity QED regime. By carefully engineering the fundamental mode and the high-order mode of the micropillars, we have realized both up-converted and down-converted single-photon emission under the dual-resonance condition. In particular, the intra-dot transitions between the X and the CX in the down-conversion process effectively suppress the carrier recapturing process by the defects states in semiconductor and therefore improve the single-photon purity of the emission. We further show that the excitation efficiency under dual-resonance conditions can be greatly improved by utilizing the vectorial excitation beams with the same polarization states as the high-order cavity modes^23,24^.

## Results

**Fig. 1:**
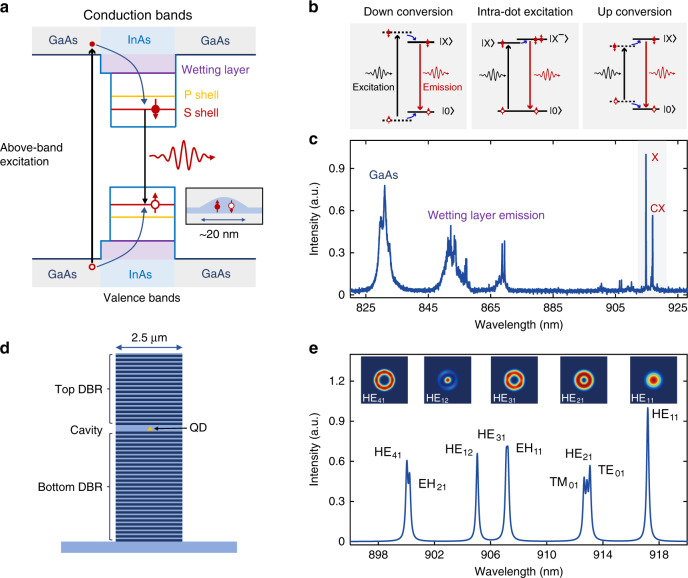
QD-micropillar system. **a** Schematic of the energy diagram of InAs QD embedded in GaAs matrix. The quantum confinement of carriers in the QD results in discrete energy levels. **b** Different excitation schemes for triggering the single-photon emissions from exciton states in QDs used in the work. All the excitation methods can be improved by the optical resonances provided from microcavities. The black and red lines represent the optical excitation and photon emission processes respectively while the blue lines denote the non-radiative process. **c** Representative spectrum of a single QD under the low-power above-band excitation. **d** Schematic of the coupled QD-micropillar system. **e** The calculated radiation spectrum and the mode profiles of the micropillar cavity with a diameter of 2.5 μm

We use single InAs QDs embedded in a GaAs matrix as quantum emitters^25,26^, as schematically shown in Fig. 1a. Due to the quantum confinement of carriers at the nanoscale, the QD exhibits atomic-like discrete energy levels, such as S-shell and P-shell. Carriers can be excited by using a laser with an energy higher than the bandgap of GaAs, referred as to above-band excitation (denoted by the thick black arrow). The created carriers in the GaAs material then relax to the lowest excited states of the QD via electron-phonon scattering before the radiative recombination process of single-photon emissions. The longitudinal optical or acoustic phonons in the solid-state provide an additional degree of freedom over the atomic systems to excite the QDs via both down- and up-conversion processes^27–30^, as shown in Fig. 1b. More interestingly, the transition between exciton states with different charge configurations (referred as to intra-dot excitation in this work) can also be utilized to trigger the radiative process as recently demonstrated in two-dimensional semiconductor^31^. A representative emission spectrum of a single InAs QD under the low-power above-band excitation is presented in Fig. 1c, exhibiting a broadband GaAs band edge emission, a wetting layer emission, and sharp X and CX lines. To build a coupled QD-micropillar system, the single InAs QD is embedded in the center of a semiconductor planar cavity consisting of a λ-thick GaAs spacer sandwiched by GaAs/Al_0.9_Ga_0.1_As distributed Bragg reflectors (DBR) with 18(26) top (bottom) pairs grown via molecular beam epitaxy. Micropillars are then fabricated from the planar cavity in order to reduce the cavity mode volume for further enhancing photon-exciton interaction, as schematically shown in Fig. 1d. Micropillar supports a series of cavity modes with sharp resonances over a broad bandwidth^32,33^. In Fig. 1e, the mode family of a 2.5 μm micropillar with the planar cavity resonance at 920 nm is calculated by the finite difference time domain (FDTD) simulation with the insets representing the intensity profiles of a few representative modes. Among all the cavity modes, the fundamental mode HE_11_ exhibits the highest Q-factor, lowest mode volume, and near Gaussian far-field pattern, which is widely used for building high-performance single-photon sources via the cavity QED effect^17,34–36^.

**Fig. 2:**
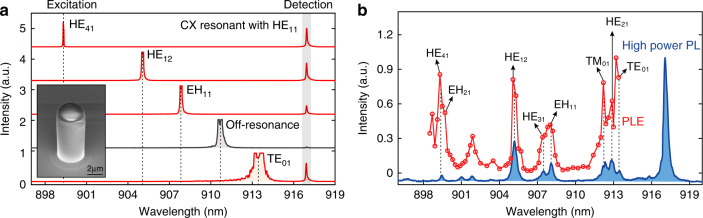
Identification of the cavity modes of the micropillar. **a** PL spectra for the QD-micropillar excited by a laser with varied wavelengths. The CX emission is resonant and therefore enhanced by the HE_11_ mode. Appreciable CX emission is observed when the excitation laser is resonant with one of the high-order cavity modes (red spectrum). In the off-resonance condition, the CX emission intensity is negligible (black spectrum). Inset, SEM image of the fabricated micropillar. **b** PLE spectrum (red) for the CX state resonant with the HE_11_ mode and the PL spectrum (blue) of the QD-cavity system under high-power above-band excitation conditions. The high-power PL is shifted slightly in y direction for clarity

The deterministically coupled QD-micropillars are fabricated by using the fluorescence imaging technique^35,37,38^ which ensures that the single QDs are both spectrally and spatially matched with the fundamental cavity mode (HE_11_) of the micropillar. The calculated collection efficiency for this mode is up to 86.6%. We first identify the mode family of the micropillar by scanning the excitation laser through all the high-order cavity modes and monitoring the emission from the CX state in resonance with the fundamental cavity mode (HE_11_). The power of the excitation laser is kept at a constant level before the objective lens to avoid saturating the QD. As long as the excitation laser is tuned to one of the high-order cavity modes, bright CX emission is observed (red spectra). On the contrary, the CX emission is barely detectable when the excitation laser is detuned from any of the high-order cavity modes, as shown by the black spectrum in Fig. 2a. The identifications of the high-order cavity modes under the dual-resonance condition are further quantified via photoluminescence excitation (PLE) spectrum (red points) in Fig. 2b in which the emission intensity of the CX state is plotted as a function of the wavelength of the excitation laser. The saturation powers and the saturated emission intensities via different cavity excitations are systematically investigated, as presented in the supplementary information. The high-order cavity modes are confirmed by another independent experiment in which cavity modes are mapped from the PL spectrum (blue curve) under high-power above-band excitation. In such an excitation scenario, the multi-exciton states and the exciton-wetting-layer hybrid states are populated, serving as a broadband internal light source to efficiently probe all the cavity modes^39^. The experimentally measured radiation spectrum is in good agreement with the calculated one presented in Fig. 1e.

**Fig. 3:**
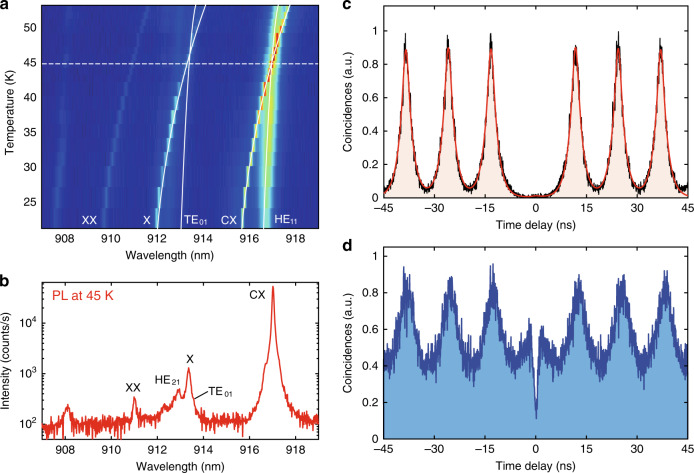
Dual-resonance enhanced X-CX transition for the highly pure single-photon emission. **a** Temperature-dependent PL mapping of a QD coupled to a 2.5 μm diameter micropillar cavity, the white line is guide for the eyes. **b** Log scaled line cut of PL mapping in (**a**) from a QD obtained under above-band (780 nm) excitation at temperature of 45 K. At this temperature, the CX is tuned into resonance of the HE_11_ mode and X is resonant with TE_01_ mode. **c**, **d** Hanbury Brown and Twiss (HBT) measurements of single-photon purity for the dual-resonance enhanced intra-dot excitation and the above-band excitation. Strong suppression of the carrier recapturing process is observed via intra-dot excitation, leading to a background-free g^(2)^(0) value as low as 0.01

We further show, for the first time, that the intra-dot transition process between the exciton states in the S-shell of a QD can be facilitated under the dual-resonance condition, as recently demonstrated in two-dimensional semiconductor^31^. Such a process enables the high-purity single-photon emission due to the absence of carrier recapturing process by the defect state in the semiconductor. Due to the Coulomb interactions of the confined carriers, there is a slight energy shift between the X and CX^40,41^. The intra-dot transition is implemented by tuning the excitation laser to match the energy of X state (913.4 nm) and monitoring the emission from the CX state (917.02 nm). The resonance condition can be reached simultaneously for both X and CX by tuning the temperature of the sample. When changing the temperature of the sample, the shift of QD energy is faster than that of the cavity mode, which brings the QD and the cavity into the resonance, as shown by the temperature-dependent spectra in Fig. 3a. At 45 K, the X and CX states are simultaneously resonant to the TE_01_ and HE_11_ modes respectively, as presented in Fig. 3b. The photon statistics of the CX emission is examined by the Hanbury-Brown-Twiss (HBT) interferometer and the coincidence histogram of the second-order correlation function is presented in Fig. 3c. Under the dual-resonance enhanced intra-dot excitation, the coincidence event at the zero delay is almost vanishing with a near-zero background across the whole histogram, indicating the generation of single-photon emission with high purity from the CX state. For comparison, the photon statistics of the CX emission under the above-band excitation condition (pumping at 780 nm) are presented in Fig. 3d. In such a case, the high-energy carriers are generated in the GaAs within the laser spot and then relax into the QDs with low-energy levels via the interactions with phonons. During the relaxation process, the carriers could be recaptured by the middle-energy level defect states, which results in a significant background for the second-order correlation^42–44^. We note that although the excitations via other high-order cavity modes with energy below the GaAs bandgap or wetting lay can also significantly suppress the carrier recapturing process, only the HBT result obtained under dual-resonance enhanced intra-dot excitation (excited from neutral exciton (913.4 nm)) shows nearly perfect suppression of background at zero delay, as presented in Fig. S1b of Supplementary Information.

**Fig. 4:**
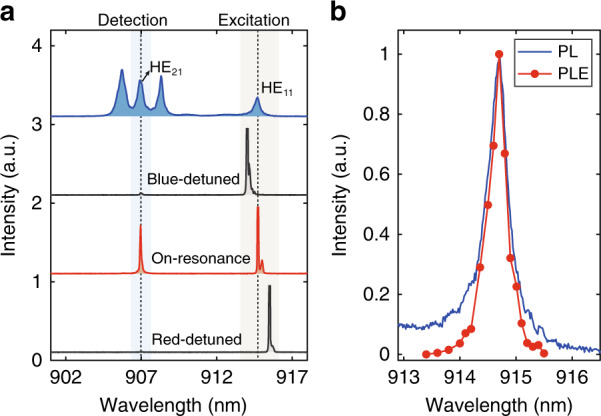
Dual resonances enhanced up-converted excitation. **a** High-power above-band PL spectrum of a pillar with a diameter of 2 μm (blue) and low-power PL spectra of a QD excited under dual resonances (red) and single resonance conditions (black). **b** Normalized high-power PL spectrum of HE_11_ mode (blue) and PLE spectrum of the QD acquired by sweeping the excitation laser through HE_11_ mode and detecting the QD emission intensity (red), the power of excitation laser is kept constant for PLE measurement

Comparing to the down-conversion process, the up-conversion process is more challenging since it extracts energy out of the system. In the up-conversion process, one low-energy photon in the excitation laser absorbs an acoustic phonon and results in the emission of a single photon from the exciton state. Such processes have been recently employed to cool the mechanical motions of a micro-resonator to its quantum ground state^45,46^ or bulk temperature of semiconductors^47–49^, showing great potential in exploring fundamental quantum physics and exploiting novel optical refrigeration methods for nanophotonic devices. As opposed to the dual-resonance enhanced down-conversion process, the up-conversion process utilizes the fundamental mode to boost the optical excitation and the high-order cavity modes to enhance the photon emission. As shown in Fig. 4a, the PL spectra of another micropillar with a diameter of 2 μm under the detuned conditions (41 K) are presented. Under the high-power above-band excitation condition (blue spectrum), the cavity modes of HE_11_, TE_01_, HE_21_, and TM_01_ are clearly identified. The excitation laser is then scanned across the HE_11_ mode (914.7 nm) to excite the X state resonant with the HE_21_ mode (906.95 nm). Under the dual-resonance condition (red spectrum), bright X state emission is observed. Such emission is nearly vanishing once the laser is either slightly red or blue-detuned from the HE_11_ mode (black spectra). The PLE spectrum of the HE_11_ mode matches excellently with the cavity resonance observed in the high-power PL spectrum under the above-band excitation condition as shown in Fig. 4b, indicating that the up-conversion process is enhanced by the cavity. Further developments along this direction may result in the realizations of reduced electron-phonon interactions in the system and even the development of optical refrigeration for single QDs.

**Fig. 5:**
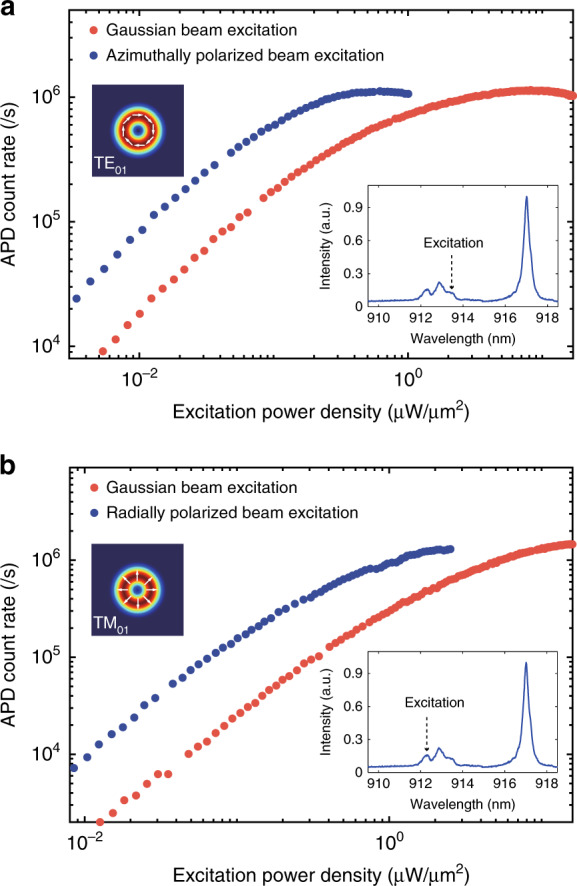
Power dependence of QD emission excited by vectorial beams. The CX is resonant with HE_11_ mode, and excited via the azimuthally polarized TE_01_ mode (blue) (**a**) and the radially polarized TM_01_ mode (blue) (**b**) to match the polarization states of the high-order cavity modes. For comparison, a linear polarized Gaussian beam excitation (red) is implemented

Finally, we show that the efficiency of the down-conversion process under the dual resonances condition can be further improved by engineering the polarization state of the excitation beam^50^. Instead of the linearly polarized HE_11_ mode, the high-order cavity modes exhibit vectorial polarizations, e.g., the TE_01_ mode is azimuthally polarized while TM_01_ mode is radially polarized. To prepare the vectorial beams, a vortex retarder with *m* = 1 (VR1-905, LBTEK) is used, by which the incident Gaussian beam with polarization perpendicular (parallel) to the fast axis of the vortex retarder is modulated to azimuthally polarized (radially polarized) beam. Figure 5a shows the single-photon emission intensity of single photons from the CX state as a function of excitation laser power at 913.5 nm (resonant with the TE_01_ mode) with different states of polarization. While the emission intensity at the saturation power is the same, the excitation power required to saturate the QD is reduced from 7.1 μW/μm^2^ to 0.4 μW/μm^2^ by switching the excitation laser from a linearly polarized Gaussian beam to a radially polarized vortex beam. Similar behavior can be observed for the excitation via the TM_01_ mode, as shown in Fig. 5b, in which the saturation power is reduced by a factor of 7 by using the azimuthally polarized excitation beam.

## Discussion

To conclude, we show versatile accessing of dual-resonance conditions for enhancing the light-matter interactions in QD-micropillar devices operating in the cavity QED regime. The cavity mode family is independently identified in both the PLE measurement and the high-power PL spectrum under the above-band excitation condition. By exploiting the intra-dot excitation under the dual-resonance condition, the single-photon purity of emitted photons is greatly improved compared to the above-band excitation condition due to the suppression of the carrier recapture process by the defects in the semiconductor. The up-converted emission is further demonstrated by using excitation via fundamental cavity mode and emission at the high-order cavity resonance. Such a process could be used to engineer electron-phonon interactions and optical refrigeration of single QDs. By engineering the polarization state of the excitation laser beam, the excitation efficiency can be further boosted. Moving forward, it is highly desirable to systematically investigate the coherence properties, e.g., linewidth and indistinguishability, of the single-photon emissions under the dual-resonance conditions for potentially advancing the photonic quantum technology. The QD-micropillar system under dual-resonance conditions may serve as an ideal platform in solid-state for investigating light-matter interaction in the quantum regime and developing integrated quantum photonic devices with high performances.

## Materials and methods

### Sample growth

The investigated sample consists of a single layer of low density In(Ga)As QDs grown via molecular beam epitaxy and located at the center of a λ-thick GaAs cavity surrounded by two Al_0.9_Ga_0.1_As/GaAs Bragg mirrors with 18(26) pairs. The density of self-assembled InAs quantum dots varies continuously along the wafer by stopping the rotation of the substrate during InAs deposition. In our experiment, a density of about 10^8^ cm^−2^ was chosen for photoluminescence imaging.

### Micropillar fabrication

The mark arrays with 10 nm Ti and 100 nm Au are first formed on the surface of the sample by the standard lift-off process, then the location of the QDs are acquired by optical positioning technique. Next, the sample is spin coated with a negative tone electron beam resist (HSQ fox16); The resist is exposed using a VISTEC EBPG5000 ES PLUS electron-beam lithography (EBL) system at 100 kV; Followed by the exposure and development process, the mask pattern of the pillar with a certain diameter is transferred into the sample via an inductively coupled plasma reactive ion etching system (ICP-RIE, Oxford Instrument Plasmalab System 100 ICP180).

### Optical measurements

An optical microscopy cryostat (Montana, *T* = 4 K-300 K) mounted on a motorized positioning system with piezo-electric actuators is used for optical measurements. A wavelength-tunable continuous-wave Ti:Sapphire laser (M squared) is used to excite the QDs. The laser beam was focused onto a selected QD-micropillar device with the laser spot of ~1.5 μm. To remove reflected excitation light, a tunable 920 nm Band-pass filter with a bandwidth of 1 nm is inserted in front of the spectrometer. The auto-correlation measurements are taken out using typical Hanbury Brown and Twiss (HBT)-type setup. The azimuthally polarized and the radially polarized beam are acquired by passing the linear polarized Gaussian laser beam through the vortex retarder with *m* = 1.

## Supplementary information

Supplementary Information for
