# Supplementary material for: Dual-resonance enhanced quantum light-matter interactions in deterministically coupled quantum-dot-micropillars
Source: Light Sci Appl. 2021 Jul 29;10:158. doi: 10.1038/s41377-021-00604-8 (PMC8322385; doi:10.1038/s41377-021-00604-8)
Supplement: Supplementary file 1 — Supplementary Information for [file 41377_2021_604_MOESM1_ESM.docx]

**Supplementary Information for**

**Dual-Resonance Enhanced Quantum Light-Matter Interactions in Deterministically Coupled Quantum-Dot-Micropillars**

Shunfa Liu^1,*^, Yuming Wei^1,*^, Xueshi Li^1^, Ying Yu^1,†^, Jin Liu^1,‡^, Siyuan Yu^1,2^, and Xuehua Wang^1^

^1^ State Key Laboratory of Optoelectronic Materials and Technologies, School of Physics, School of Electronics and Information Technology, Sun Yat-sen University, Guangzhou 510275, China

^2^ Photonics Group, Merchant Venturers School of Engineering, University of Bristol, Bristol BS8 1UB, United Kingdom

* These authors contributed equally

† yuying26@mail.sysu.edu.cn

‡ liujin23@mail.sysu.edu.cn

1. **Comparison between HBT results**

In figure 3 of the main text, we have presented the HBT results measured under dual-resonance enhanced intra-dot excitation (913.4 nm) and above-band excitation (780 nm) to demonstrate the advantage of intra-dot excitation between X-CX transition for suppressing the recapture process. Here, we compare this with the results obtained under the condition of HE_21_ (912.93 nm) cavity resonant excitation. The excitation wavelength is marked in fig. S1(a), and corresponding HBT results are presented in fig.S1(b). As shown, although all of those excitation conditions are enhanced by cavity resonance, only the result obtained under DRE condition (excited from neutral exciton (913.4 nm)) shows nearly perfect suppression of background at zero delay.

**Fig. S1** (a) log scaled PL of the QD at 45 K used in main text for investigating cavity enhanced intra-dot excitation process. (b) HBT results taken under HE_21_ (912.93 nm) , dual-resonance enhanced intra-dot excitation (913.4 nm).

1. **Power dependence of QD under different cavity resonance conditions**

In addition to the PLE spectra presented in fig 2b of main text, which shows strong cavity dependence, we have also performed power dependent measurements under different excitation conditions, as shown in Fig.S2 and Table.S1. The results presented in fig.S2(b) are measured under cavity resonance conditions (HE_41_ mode at 899.4 nm and TM_01_ mode at 912.2 nm) and non-resonance conditions (909 nm and 915 nm) respectively. As can be seen, the saturation power is significantly reduced by cavity enhancement. Without the cavity enhancements, the QDs are not saturated even under very high-power for a laser with an energy lower than the bandgap of GaAs (e.g., 909 nm and 915 nm). Once the excitation laser is resonant with any of the high-order cavity modes, the QDs can be saturated and the saturation power seems to be lower when the excitation laser is moving towards the emission wavelength. Comparison of saturation power and emission intensity under different excitation conditions are presented in **Table.S1**.

**Fig. S2** (a) Cavity mode spectra of a micropillar with diameter of 2.5 μm obtained under high power above-band excitation. (b) Power dependence of the QD emission under different excitation conditions with varied wavelength.

**Table. S1**

Comparison of saturation power and emission intensity under different excitation conditions.

| Excitation wavelength (nm) | 899.4 | 905.2 | 908.1 | 912.2 | 912.9 | 913.4 |
| --- | --- | --- | --- | --- | --- | --- |
| Mode index | HE_41_ | HE_12_ | EH_11_ | TM_01_ | HE_21_ | TE_01_ |
| Saturated APD count rate (MHz) | 1.5 | 1.46 | 1.3 | 1.5 | 1.6 | 1.67 |
| Saturation power density (μW/μm^2^) | 59.4 | 70.5 | 45.3 | 45.2 | 19.2 | 7.1 |
